# Bridging data gaps in pregnancy outcomes: a four-year review of gestational Age records and delivery trends in Kilimanjaro region, Tanzania

**DOI:** 10.3389/frph.2026.1720135

**Published:** 2026-02-26

**Authors:** Agnes Msoka, Allen Lyimo, Gustav Nkya, Potina Zongollo, Godrule Lyimo, Jairy N. Khanga, Fatina Rashid, Modesta Mitao, Martha Oshosen, Blandina Theophil Mmbaga

**Affiliations:** 1 Kilimanjaro Clinical Research Institute (KCRI), Moshi, Tanzania; 2 KCMC University, Moshi Urban, Tanzania; 3 Kilimanjaro Christian Medical Centre, Moshi Urban, Tanzania; 4 Kilimanjaro Regional Office, Moshi, Tanzania

**Keywords:** data quality, delivery trends, gestational age, maternal records, preterm birth, Tanzania

## Abstract

**Introduction:**

Accurate gestational age (GA) documentation and reliable delivery data are essential for guiding clinical decision-making, classifying preterm and term births, and informing maternal health planning. In Tanzania, inconsistent GA recording and variable facility-based delivery patterns hinder effective monitoring of maternal and newborn outcomes. This study reviewed four years of delivery data to assess the completeness of GA documentation and explore delivery trends across urban and rural facilities in the Kilimanjaro Region.

**Methods:**

A retrospective cross-sectional trend analysis was conducted using delivery records from 2019 to 2022 across five health facilities. Maternal demographics, GA at delivery, and delivery outcomes were extracted. Completeness of GA documentation was assessed, and logistic regression was used to examine factors associated with (1) complete GA documentation and (2) delivery outcomes (preterm vs. term).

**Results:**

A total of 1,656 delivery records were reviewed. GA was documented in 78.4% of records, with higher completeness in urban (82.6%) than rural (69.8%) facilities. Institutional deliveries increased over the four years in both settings. Preterm births accounted for 12.3% of all deliveries, with higher prevalence in rural areas. In adjusted analyses, GA completeness was significantly associated with urban facility type (aOR = 1.74; 95% CI: 1.32–2.29), higher parity (aOR = 1.41; 95% CI: 1.05–1.90), and year of delivery (aOR = 1.26 per year; 95% CI: 1.11–1.43). Preterm birth was significantly associated with incomplete GA documentation (aOR = 2.08; 95% CI: 1.32–3.27).

**Conclusion:**

Delivery rates increased over time, but persistent gaps in GA documentation limit the accuracy of pregnancy classification and reporting. Improving documentation practices—particularly in rural facilities—is essential to strengthen maternal health data systems and inform evidence-based decision-making.

## Background

Gestational age (GA) assessment and facility-based delivery trends are essential indicators for monitoring maternal and newborn health outcomes. Accurate GA documentation enables proper classification of pregnancies, timely identification of preterm or post-term births, and informed clinical decision-making during labour and delivery (1, 2). Facility delivery rates serve as a proxy for access to skilled birth attendance, which significantly reduces maternal and neonatal morbidity and mortality (3, 4, 23, 37).

Despite national improvements in maternal health services, Tanzania continues to experience persistent gaps in accurate GA recording and variability in institutional delivery rates across regions (5, 6, 29, 30, 35). Inconsistent documentation—particularly in lower-level facilities—contributes to misclassification of deliveries, poor outcome tracking, and delays in initiating timely interventions such as emergency obstetric care and newborn resuscitation (7, 8).

Moreover, although antenatal care remains an important entry point for maternal health monitoring, gaps in recordkeeping during pregnancy ultimately affect the reliability of delivery data. Poorly documented GA at ANC limits the ability to predict expected delivery dates, assess risk, or plan for facility delivery (9, 24, 33, 35). Strengthening GA documentation throughout pregnancy, particularly at delivery, is therefore critical.

To date, no study in the Kilimanjaro Region has systematically examined delivery trends in parallel with the quality of gestational age (GA) documentation over multiple years. This knowledge gap constrains evidence-based planning at both facility and regional levels. The present study addresses this limitation by analysing four years of delivery records and evaluating the completeness and accuracy of GA documentation across urban and rural health facilities. This write up of the manuscript forms part of the multi-country project “*Antenatal Care Attendance Across Gestational Age Windows: Get Ready, Get Real,”* implemented in Bangladesh, Nepal, Nigeria, Tanzania, The Gambia, and Uganda.

## Objectives

- To determine the completeness and consistency of gestational age documentation in delivery records across five health facilities from 2019 to 2022.
- To analyse the association between gestational age at delivery and maternal/neonatal outcomes using regression analysis.
- To identify facility-level and maternal factors associated with incomplete GA documentation.
- To propose data-driven strategies for improving the accuracy and completeness of GA documentation in delivery records.

## Methods

### Study design

A retrospective cross-sectional trend analysis was conducted using delivery records from 2019 to 2022.

### Study setting

The study was conducted in five health facilities in Moshi Municipal, Rombo, and Mwanga districts of the Kilimanjaro Region. Facilities included three urban and two rural sites offering routine ANC and delivery services.

### Study population

The study population comprised all women who delivered in the selected facilities between 2019 and 2022. Delivery records with GA information and outcome data were eligible.

### Inclusion criteria

- ANC records of women who attended ANC and delivered at the five selected facilities.
- Records with clearly documented gestational age at the time of ANC visit or delivery.
- Records dated between January 2019 and December 2022.
- Records with documented delivery outcomes.

### Exclusion criteria

- Records with illegible, inconsistent, or contradictory information that could not be validated.
- Records lacking essential identifiers needed for linking ANC and delivery information.
- Records duplicated across registries.
- Records for women who were referred out before delivery (if applicable), as their outcomes were not available.
- Records from outside the selected facilities even if they fall within the study dates

### Data collection procedures

#### Ethical clearance

Ethical approval was obtained from the Kilimanjaro Christian Medical Centre (KCMC) Research Committee (Ref: 2643) and the National Institute for Medical Research (NIMR) in Tanzania (Ref: NIMR/HQ/R.Sa/Vol.IX/4480). The study also adhered to the principles of the World Medical Association Declaration of Helsinki.

### Statistical analysis

Data analysis was conducted using STATA version 16. Descriptive statistics were first generated to summarize the characteristics of the study population. Categorical variables were presented as frequencies and percentages, while continuous variables were summarised using means and standard deviations. Additional descriptive outputs included an assessment of annual trends in the number of recorded deliveries and the completeness of gestational age (GA) documentation over the four-year period.

For analytical assessment, two logistic regression models were applied. Table 1 (Model 1) examined factors independently associated with *complete GA documentation*. Explanatory variables included facility type (urban or rural), maternal age category, parity, year of delivery, and antenatal care (ANC) attendance patterns. Table 2 (Model 2) assessed determinants of *preterm birth*, defined as delivery before 37 completed weeks of gestation. The independent variables for this model were GA documentation completeness, maternal age, facility type, parity, and year of delivery. Statistical significance was defined as *p* < 0.05 for all analyses.

**Table 1:** (Model 1) Factors associated with complete GA documentation.

Variable	aOR	95% CI	*p*-value
Urban facility	1.74	1.32–2.29	<0.001
Parity ≥2	1.41	1.05–1.90	0.022
Year of delivery	1.26	1.11–1.43	<0.001

**Table 2:** (Model 2) Factors associated with preterm birth (<37 wks).

Variable	aOR	95% CI	*p*-value
Incomplete GA documentation	2.08	1.32–3.27	0.002
Rural facility	1.47	1.02–2.12	0.039

## Results

This section presents the key findings from the analysis of four years of delivery records and gestational age documentation across urban and rural health facilities in the Kilimanjaro Region. The results are organised into three main areas: maternal demographic characteristics, delivery trends with completeness of gestational age records, and regression analyses examining predictors of complete GA documentation and preterm birth.

### Maternal characteristics

A total of 1,656 women were included in the analysis (Table 3). The largest proportion were aged 25–29 years (30.5%), followed by those aged 20–24 years (24.9%). Adolescents (<20 years) accounted for 8.1% of the sample, while 14.1% were aged 35 years or above. Parity distribution showed that nearly one-third of the women were primiparous (31.9%), while 42.4% had one to two previous births and 25.7% had three or more. Most deliveries occurred in urban facilities (69.3%), compared with 30.7% in rural settings. This revised table aligns with reviewer comments by focusing on participant characteristics rather than facility descriptors.

**Table 3:** Maternal demographic nncharacteristics (*N* = 1,656 partcipants).

Participants characteristic	Category	*n* (%)
Age (years)	<20	134 (8.1)
	20–24	412 (24.9)
	25–29	506 (30.5)
	30–34	371 (22.4)
	≥35	233 (14.1)
Parity	0	528 (31.9)
	1–2	702 (42.4)
	≥3	426 (25.7)
Facility Type	Urban	1,148 (69.3)
	Rural	508 (30.7)

### Delivery trends and completeness of GA records

Table 4 presents delivery distributions and missing GA records across rural and urban facilities from 2019 to 2022. The number of recorded deliveries increased steadily in both settings, with rural deliveries rising from 1,707 in 2019 to 6,793 in 2022, and urban deliveries increasing from 2,586 to 6,863 during the same period.

**Table 4:** Distribution of delivery records and missing data in Rural-urban: 2019–2022.

Year	Setting	Deliveries *n* (%)	Missing GA records *n* (%)
2019	Rural	1,707 (4.9)	309 (2.2)
	Urban	2,586 (7.5)	2,162 (15.0)
2020	Rural	3,167 (9.2)	369 (2.6)
	Urban	3,674 (10.6)	1,699 (12.0)
2021	Rural	4,454 (12.9)	559 (4.0)
	Urban	5,357 (15.5)	3,040 (21.0)
2022	Rural	6,793 (19.6)	74 (0.5)
	Urban	6,863 (19.8)	5,881 (41.7)

Despite increasing delivery volumes, the burden of missing GA records varied substantially by year and setting. Rural facilities showed a sharp reduction in missing GA records by 2022 (0.5%), while urban settings reported persistently high proportions of missing data, peaking at 41.7% in 2022. These discrepancies highlight ongoing challenges in accurate GA documentation, especially in high-volume urban facilities.

### Regression analysis

#### Model 1: factors associated with complete GA documentation

Several factors were significantly associated with improved GA documentation. Deliveries occurring in urban facilities had higher odds of complete GA recording (aOR 1.74; 95% CI 1.32–2.29; *p* < 0.001). Women with parity ≥2 were also more likely to have complete GA documentation (aOR 1.41; 95% CI 1.05–1.90; *p* = 0.022). Additionally, the year of delivery showed a positive association (aOR 1.26; 95% CI 1.11–1.43; *p* < 0.001), indicating gradual improvement over time.

#### Model 2: factors associated with preterm birth

Incomplete GA documentation emerged as a strong predictor of preterm birth (aOR 2.08; 95% CI 1.32–3.27; *p* = 0.002), suggesting potential misclassification of gestational age or challenges in clinical dating. Deliveries in rural facilities were also associated with higher odds of preterm birth (aOR 1.47; 95% CI 1.02–2.12; *p* = 0.039), pointing to possible differences in maternal risk profiles, care-seeking patterns, or provider capacity.

The analysis of four years of delivery records and gestational age documentation in Kilimanjaro Region revealed important demographic, service-delivery and clinical patterns. The study population consisted largely of women aged 25–29 years and those with one to two previous births, with most delivering in urban facilities. Delivery volumes increased consistently across both rural and urban settings from 2019 to 2022, reflecting either higher service utilisation or improved reporting; however, the completeness of gestational age documentation did not improve uniformly. Rural facilities demonstrated remarkable progress, reducing missing GA records to almost zero by 2022, while urban facilities experienced persistently high and worsening levels of missing documentation, likely driven by high patient load and system inefficiencies. Regression findings further highlighted disparities in documentation quality and birth outcomes. Urban facility deliveries, higher parity, and later calendar years were associated with better GA recording, indicating gradual system strengthening. Conversely, incomplete GA documentation significantly increased the likelihood of preterm birth, suggesting potential misclassification or inadequate clinical assessment when GA is not properly documented. Additionally, women delivering in rural settings had higher odds of preterm birth, which may reflect differences in maternal risk factors, access to timely care, or facility capacity. Overall, the results underscore substantial gaps in GA documentation—particularly in urban facilities—and highlight the link between documentation quality and accurate identification of preterm births.

## Discussion

The study provides important insights into delivery patterns and documentation practices over a four-year period in the Kilimanjaro Region. While institutional deliveries have steadily increased and some improvements in record-keeping were observed, significant gaps—particularly in gestational age (GA) documentation—persist. These gaps limit the accuracy of birth classification, affect reporting of preterm births, and hinder effective clinical decision-making. The disparities identified between urban and rural facilities, alongside broader systemic challenges such as limited ultrasound access and inconsistent training, highlight ongoing inequities in maternal health service delivery (25, 27, 28, 36). Strengthening documentation systems, expanding digital health tools, and improving GA assessment capacity remain essential for enhancing data quality and ensuring better maternal and neonatal outcomes.

The four-year analysis revealed rising institutional deliveries and gradual improvements in documentation practices, but also highlighted persistent gaps—particularly in gestational age (GA) recording—that undermine accurate classification of births and reporting of outcomes (32). Our finding that GA was more completely documented in urban facilities aligns with prior research showing that better staffing, access to ultrasound, and stronger data systems contribute to higher-quality maternal health records (1, 10, 11).

The observed 12.3% preterm birth rate is consistent with national estimates for Tanzania (4, 6), but the strong association between incomplete GA documentation and preterm classification suggests possible misclassification (26). This is a known challenge in low-resource settings relying primarily on last menstrual period (LMP) rather than early ultrasound for GA estimation (1, 2). Misclassification may lead to under- or over-reporting of preterm births, which has implications for neonatal care and national reporting.

Persistent documentation gaps in rural facilities reflect broader systemic challenges, including heavy workload, inconsistent training on GA estimation, limited ultrasound access, and poor archiving or data review systems (5, 7, 10, 29, 31). Studies in Sub-Saharan Africa similarly show that rural facilities often face infrastructure and staffing limitations that hinder routine data quality (8, 12, 13).

Improving GA documentation is critical not only for accurate reporting but also for clinical decision-making, such as identifying late initiation of antenatal care, high-risk pregnancies, planning timely interventions, and informing health system readiness (3, 14, 15, 24, 34, 35, 37). Digital health tools, including electronic clinical decision support systems, have demonstrated potential to enhance antenatal and delivery record quality, especially when combined with staff training and regular data quality audits (10, 11, 16, 17).

The urban–rural disparities observed in our study also echo findings from other studies in Tanzania and similar settings, which highlight inequities in access to quality maternal care and the impact of health system factors on maternal outcomes (6, 9, 18). Strengthening rural health systems through infrastructure improvements, targeted staffing, and supportive supervision is therefore essential to ensure equitable maternal care delivery and reliable data collection (5, 19, 20).

Finally, our findings reinforce the need for routine GA training, adoption of digital maternity registers, and expanded ultrasound coverage to improve the accuracy and completeness of delivery records (10, 16, 21, 22).

## Conclusion

Delivery rates increased in both urban and rural facilities from 2019 to 2022, reflecting improved access to skilled care. However, incomplete GA documentation remains a major barrier to accurate reporting and risk assessment. Strengthening documentation systems—especially in rural areas—is critical to improving the reliability of maternal health data and informing policy and practice.

## Recommendations

To improve gestational age (GA) assessment and delivery record quality, several key recommendations are proposed. Routine training on GA assessment and documentation should be introduced for healthcare providers, alongside the implementation of digital maternity registers and decision-support tools to streamline data collection. Expanding ultrasound coverage, particularly for early pregnancy dating, is essential to enhance accuracy. Additionally, supportive supervision and regular data quality audits should be strengthened to ensure consistency and reliability of records. Finally, the capacity of rural health systems should be enhanced through targeted staffing and infrastructure support to address disparities in service delivery.

## Data Availability

The original contributions presented in the study are included in the article/Supplementary Material, further inquiries can be directed to the corresponding author.

## References

[1] Pernille NN Wang C Reilly JJ Sanga G Sørensen L Manongi R Agreement between last menstrual period and ultrasound-estimated gestational age among Tanzanian women. Int J Nurs Midwifery. (2021) 13(3):26–34. 10.5897/IJNM2021.0465

[2] Deputy NP Nguyen PH Pham H Nguyen S Neufeld L Martorell R Validity of gestational age estimates by last menstrual period and neonatal examination compared to ultrasound in Vietnam. BMC Pregnancy Childbirth. (2017) 17(1):1–10. 10.1186/s12884-016-1192-5 28077098 PMC5225544

[3] World Health Organization. Improving quality of care for maternal, newborn and child health. World Health Organization (2022). Available online at: https://www.who.int/publications/i/item/WHO-MCA-22.01 (Accessed February 9, 2026).

[4] Soibi-Harry AP Osanyin GE Okunade KS Afolabi BB. Neonatal outcomes of early-term vs. late-term births in Lagos, Nigeria. Cureus. (2024) 16(4):e57833. 10.7759/cureus.57833 38721170 PMC11078171

[5] Massenga J Jeremiah K Kitinya W Kim YM van Roosmalen J van den Akker T. Receiving antenatal care components and associated factors in northwestern Tanzania. PLoS One. (2023) 18(4):e0284049. 10.1371/journal.pone.0284049 37040366 PMC10089348

[6] Kasagama E Todd J Renju J. Factors associated with adequate antenatal care visits among pregnant women aged 15–49 years in Tanzania. BMC Pregnancy Childbirth. (2022) 22(1):1–11. 10.1186/s12884-021-04350-y 34996378 PMC8742319

[7] Ajegbile ML. Closing the gap in maternal health access and quality through targeted investments in low-resource settings. J Glob Health Rep. (2023) 7:1–5. 10.29392/001c.88917

[8] Endawkie A Kebede N Asmamaw DB Tsega Y. Predictors and number of antenatal care visits among women in sub-Saharan Africa: zero-inflated negative binomial regression. PLoS One. (2024) 19(10):e0302297. 10.1371/journal.pone.0302297 39436932 PMC11495606

[9] Rwabilimbo AG Ahmed KY Page A Ogbo FA. Trends and factors associated with antenatal care utilisation in Tanzania. Trop Med Health. (2020) 48(1):1–12. 10.1186/s41182-020-00226-7 32518496 PMC7268642

[10] van Pelt S Massar K Shields-Zeeman L de Wit JBF van der Eem L Lughata AS Development of an electronic clinical decision support system to improve antenatal care in rural Tanzania. Front Public Health. (2021) 9:645521. 10.3389/fpubh.2021.645521 34095055 PMC8172617

[11] van Pelt S van der Pijl M Ruiter RAC Ndaki PM Kilimba R Shields-Zeeman L Pregnant women's Perceptions of digital health tools in Tanzania: a qualitative study. Sex Reprod Health Matters. (2023) 31(1):2236782. 10.1080/26410397.2023.2236782 37503741 PMC10388793

[12] Mlandu C Matsena-Zingoni Z Musenge E. Trends and determinants of late antenatal care initiation in three east African countries. PLoS Glob Public Health. (2022) 2(8):1–15. 10.1371/journal.pgph.0000534 PMC10021240 36962755

[13] Lateef MA Kuupiel D Mchunu GG Pillay JD. Utilization of antenatal care and skilled birth services in sub-Saharan Africa: a systematic scoping review. Int J Environ Res Public Health. (2024) 21(4):1–18. 10.3390/ijerph21040440 PMC11050659 38673351

[14] Tunçalp Ö Pena-Rosas JP Lawrie T Bucagu M Oladapo OT Portela A WHO recommendations on antenatal care for a positive pregnancy experience. BJOG. (2017) 124(6):860–2. 10.1111/1471-0528.14599 28190290

[15] World Health Organization. WHO recommendations on antenatal care for a positive pregnancy experience: 2024 update. World Health Organization (2024). Available online at: https://www.who.int/publications/i/item/9789241549912 (Accessed February 9, 2026). 28079998

[16] Till S Mkhize M Farao J Shandu LD Muthelo L Coleman TL Digital health technologies for maternal and child health in Africa: a scoping review. J Med Internet Res. (2023) 25:e42161. 10.2196/42161 37027199 PMC10131761

[17] Misago N Habonimana D Ciza R Ndayizeye JP Kimaro JKA. A digitalized program to improve antenatal healthcare in rural Burundi: early lessons. PLoS Digital Health. (2023) 2(4):e0000133. 10.1371/journal.pdig.0000133 37068064 PMC10109494

[18] Alhassan Y Otiso L Okoth L Murray L Hemingway C Lewis JM Four antenatal care visits by four months of pregnancy and four vital tests for pregnant mothers: impact of a community–facility health systems strengthening intervention in Migori county, Kenya. BMC Pregnancy Childbirth. (2024) 24(1):1–13. 10.1186/s12884-024-06386-2 38539129 PMC10967157

[19] McGuire F. Evaluating the effects of health system policies on healthcare access in low- and middle-income countries. (Doctoral dissertation). London School of Hygiene & Tropical Medicine (2022).

[20] Ministry of Health. Digital health strategy: July 2019–June 2024. Ministry of Health, United Republic of Tanzania (2019). Available online at: https://www.moh.go.tz (Accessed February 9, 2026).

[21] Patel SJ Subbiah S Jones R Muigai F Rothschild CW Omwodo L Providing support to pregnant women through moderated WhatsApp groups: a feasibility study. mHealth. (2018) 4:14. 10.21037/mhealth.2018.04.05 29963559 PMC5994467

[22] Kibesaa SJ Kitua DW. Determinants of antenatal healthcare services utilisation in Tanzania. East Afr Health Res J. (2022) 6(2):155–61. 10.24248/eahrj.v6i2.701 36751686 PMC9887509

[23] Antenatal care – UNICEF Data. (n.d.). Available online at: https://data.unicef.org/topic/maternal-health/antenatal-care/ (Accessed December 21, 2024).

[24] Boka A Alemu M Gela D. Delayed antenatal care and associated factors among pregnant women attending health facilities in Addis Ababa, Ethiopia. Afr J Midwifery Womens Health. (2023) 17(3):1–11. 10.12968/ajmw.2022.0036

[25] Burns A DeAtley T Short SE. The maternal health of American Indian and Alaska native people: a scoping review. Soc Sci Med. (2023) 317:115584. 10.1016/j.socscimed.2022.115584 36521232 PMC9875554

[26] Chawanpaiboon S Titapant V Pooliam J. Maternal complications and risk factors associated with assisted vaginal delivery. BMC Pregnancy Childbirth. (2023) 23(1):1–9. 10.1186/s12884-023-06080-9 37884886 PMC10601252

[27] Diamond-Smith N Sudhinaraset M Montagu D. Clinical and perceived quality of care for maternal, neonatal and antenatal care in Kenya and Namibia: the service provision assessment. Reprod Health. (2016) 13(1):1–13. 10.1186/s12978-016-0208-y 27515487 PMC4981972

[28] Fernandez Turienzo C Newburn M Agyepong A Buabeng R Dignam A Abe C Addressing inequities in maternal health among socially disadvantaged communities. BMC Public Health. (2021) 21(1):1–5. 10.1186/s12889-021-10182-4 33478445 PMC7817762

[29] Jinga N Mongwenyana C Moolla A Malete G Onoya D. Reasons for late presentation for antenatal care: healthcare providers’ perspectives. BMC Health Serv Res. (2019) 19(1):1–9. 10.1186/s12913-019-4855-x 31888616 PMC6937646

[30] Kibesaa SJ Kitua DW. Determinants of antenatal healthcare services utilisation in Tanzania. J Womens Health Reprod Med. (2022) 6(2):1–9. 10.24248/eahrj.v6i2.701 PMC9887509 36751686

[31] Stringer K Turan KL McCormick B Durojaiye L Nyblade M Kempf L Barriers to engaging pregnant women in care: a qualitative study. Physiol Behav. (2017) 176:139–48. 10.1002/hep.3015028363838

[32] Kulkarni S Harvey B Prybylski D Jalloh MF. Trends in classifying vaccine hesitancy reasons in the WHO/UNICEF joint reporting form (2014–2017). Hum Vaccin Immunother. (2021) 17(7):2001–7. 10.1080/21645515.2020.1859319 33534626 PMC8189077

[33] Kumar G Choudhary TS Srivastava A Upadhyay RP Taneja S Bahl R Determinants of full antenatal care in India: analysis from national family health survey–4. BMC Pregnancy Childbirth. (2019) 19(1):327. 10.1186/s12884-019-2473-6 31488080 PMC6727513

[34] MoHCDGEC. Antenatal Care Guidelines. Tanzania: Ministry of Health (2018).

[35] Tadele F Getachew N Fentie K Amdisa D. Late initiation of antenatal care and associated factors among pregnant women in Jimma zone, Ethiopia. BMC Health Serv Res. (2022) 22(1):1–8. 10.1186/s12913-022-08055-6 35549700 PMC9097060

[36] Webster D Muller L. Urban Competitiveness Assessment in Developing-Country Regions. London: INFUD Urban Group (2000).

[37] World Health Organization. WHO recommendations on antenatal care for a positive pregnancy experience. World Health Organization (2016). Available online at: https://iris.who.int/handle/10665/250796 (Accessed February 9, 2026). 28079998

